# Machine learning algorithms for predicting atrial fibrillation using single-lead data derived from 12-lead ECGs

**DOI:** 10.3389/fcvm.2025.1612750

**Published:** 2025-10-07

**Authors:** Ji-Hoon Choi, Sung-Hee Song, Jongwoo Kim, JaeHu Jeon, KyungChang Woo, Soo Jin Cho, Seung-Jung Park, Young Keun On, Ju Youn Kim, Kyoung-Min Park

**Affiliations:** ^1^ Division of Cardiology, Department of Internal Medicine, Konkuk University Medical Center, Konkuk University School of Medicine, Seoul, Republic of Korea; ^2^ Wellysis Corp., Seoul, Republic of Korea; ^3^ MediFarmSoft Co., Ltd., Seoul, Republic of Korea; ^4^ Center for Health Promotion, Samsung Medical Center, Sungkyunkwan University School of Medicine, Seoul, Republic of Korea; ^5^ Division of Cardiology, Department of Medicine, Heart Vascular Stroke Institute, Samsung Medical Center, Sungkyunkwan University School of Medicine, Seoul, Republic of Korea

**Keywords:** atrial fibrillation, electrocardiogram, artificial intelligence, machine learning, single-lead, wearable devices, prediction

## Abstract

**Background:**

Wearable electrocardiogram (ECG) monitoring devices that utilize single-lead ECG technology have become valuable tools for identifying paroxysmal atrial fibrillation (AF). This study aimed to develop a machine learning (ML) algorithm to predict new-onset AF by training it on single-lead data extracted from 12-lead ECG recordings.

**Methods and results:**

Patients who underwent 12-lead ECG between January 2010 and December 2021 were classified into two groups based on a review of their medical records and diagnostic codes: the AF group and the normal group. An ML model was created using single-lead ECG data, excluding three augmented leads, and incorporating 60 calculated statistical variables for each of the remaining single leads. The model's performance was assessed using several metrics, including the area under the receiver operating characteristic curve (AUROC), sensitivity, specificity, accuracy, and F1 score. We trained the ML model on 248,612 ECGs collected from 106,606 patients, of whom 11,810 had definite AF. Among the single-lead machine learning models developed from each of the nine individual leads, lead I demonstrated the best performance. The AUROC of the single-lead ECG ML model using lead I was 0.801, while the AUROC of the 12-lead ECG ML model was 0.816.

**Conclusion:**

The single-lead ECG ML model has shown promise in predicting new-onset atrial fibrillation (AF), particularly with lead I. Its performance is comparable to that of the 12-lead model.

## Introduction

Atrial fibrillation (AF) is a common arrhythmia that increases the risks of stroke and heart failure and imposes a substantial healthcare burden (1–3). Although AF is traditionally diagnosed with 12-lead electrocardiograms (ECGs) or Holter monitoring, its paroxysmal nature means many cases remain undetected until complications occur. Predicting AF before clinical manifestation could enable earlier intervention and reduce adverse outcomes (4).

Recent advancements in healthcare technology have introduced single-lead electrocardiogram (ECG) monitoring devices, which are transforming the detection and management of arrhythmias (5, 6). These devices allow for continuous or event-triggered recordings, making them particularly effective in diagnosing paroxysmal episodes of AF, which are often difficult to detect (7). Nevertheless, current diagnostic strategies remain imperfect, leaving a subset of patients at risk of missed diagnoses and unclear follow-up pathways (8). Contemporary guidelines therefore recommend prolonged, non-invasive ECG-based screening in selected populations (9).

Concurrently, Artificial intelligence–enabled electrocardiography (AI-ECG) has emerged as a transformative approach for early detection and prediction of atrial fibrillation (AF), particularly within outpatient and remote-monitoring pathways (10, 11). By learning subtle signatures of atrial remodeling from routine ECGs—including single-lead tracings—AI can identify individuals at risk before AF is documented, enabling scalable screening and risk-stratified follow-up (12).

In this context, we investigate prediction from single-lead ECGs derived from standard 12-lead recordings. Specifically, we (i) evaluate how AF-prediction performance varies when each individual lead is trained separately, and (ii) compare single-lead models with our previously developed 12-lead AI model (13).

## Methods

### Collection of ECG data

All standard 12-lead ECGs obtained from patients aged 18 years and older at Samsung Medical Center between January 2010 and December 2021 were selected for this study. The ECGs were conducted using Philips ECG instruments (PageWriter TC70, TC50, TC30, and Trim III) at a sampling rate of 500 Hz. Each recording lasted 10 s and had a resolution of 5 µV. The raw data were stored in XML (Extensible Markup Language) format. Out of the 12 leads, only 9 were used in the analysis, excluding the augmented leads (aVR, aVL, and aVF), which are derived from other limb leads.

A database of ECGs was created from all collected records, which were labeled based on readings from trained physicians and cardiologists. The research protocol was approved by the Samsung Medical Center Institutional Review Board, which also granted a waiver for informed consent in accordance with our institutional ethics policy.

For external validation, 12-lead ECG data from Wonju Severance Hospital, recorded using a General Electric ECG machine, were utilized. In the ECG data from Wonju Severance Hospital, lead III data was unusable. Therefore, a total of eight leads, excluding the three augmented leads and lead III, were utilized.

### Identifying study groups

All cases underwent a thorough review of medical records and diagnostic codes. A diagnosis of definite AF was assigned only to those patients who had a documented AF ECG, whether from a 12-lead ECG or Holter monitoring, along with a confirmed AF diagnosis in medical records or diagnostic codes. All patients in this study were classified into either an AF group or a normal group based on the established criteria for definite AF. The index date for AF was defined as the earliest date on which AF was diagnosed, based on the available ECG records and medical documentation.

The exclusion criteria for this study were as follows: (1) patients who had a diagnosis of AF documented in their medical records or recorded with a diagnostic code for AF prior to the index AF ECG, (2) patients who did not have a normal sinus rhythm (NSR) ECG before the index AF ECG, (3) patients who had only one NSR ECG, (4) patients with a medical record or diagnostic code indicating AF but without an AF ECG, (5) patients with insufficient medical records to assess their medical status, and (6) patients with abnormal ECGs (defined as any ECG not labeled as NSR or within the normal range). The ECGs of all patients who were not excluded were included in this study.

### Machine learning model development

**Figure 1:**
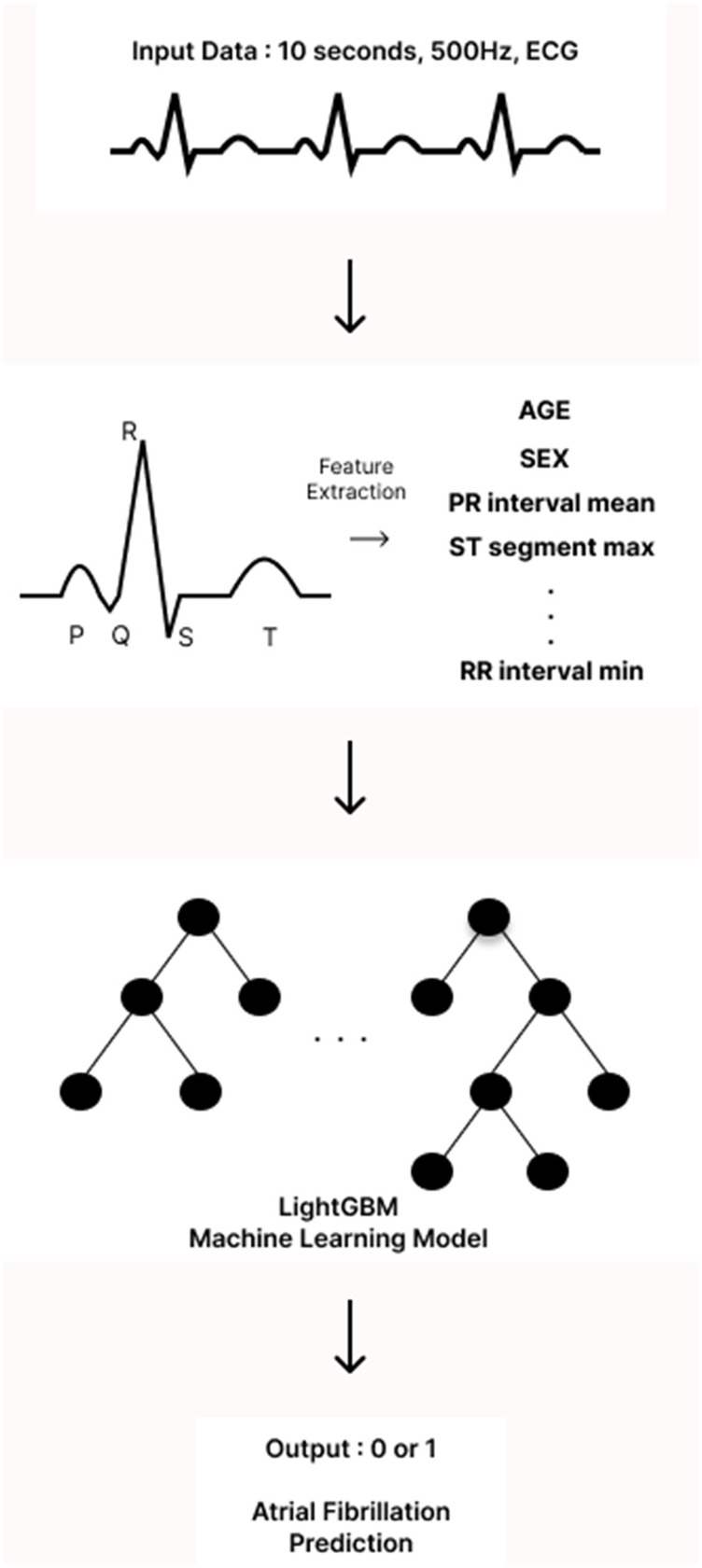
Development of a machine learning model using single-lead ECG.

The analysis of ECGs began with the identification of the onset of the *P* wave, which is the first wave of the cardiac cycle. The ECG signals were then processed using a band-pass filter (0.5–45 Hz) to eliminate unwanted artifacts, such as baseline wandering and power line interference. Next, all components of the P-QRS-T waves—specifically the peaks, intervals, and segments—were detected and located using Neurokit2, an open-source Python package designed for neurophysiological signal processing. From the extracted P-QRS-T components, we calculated descriptive statistics that served as input features for our models. In particular, we focused on the mean, minimum, maximum, and standard deviation of the peaks, intervals, segments, and durations. To better capture the shape of the *P* wave in our model, we isolated the *P* wave from the P-off to the P-on point and calculated its skewness and kurtosis. This allowed us to quantify how skewed and peaked the *P* wave was. Additionally, we computed the changes between successive peaks, intervals, and durations to capture the variability of the ECG waves. Incorporating further details, we added correlation statistics, which comprised the average of the beat-wise Pearson correlation coefficients between a template beat and other beats, as well as f-wave indices to include information related to AF. Descriptive statistics of heart rate variability were also included to provide more context from the ECG data. All features were extracted from all 12 leads of the ECG. For both single-lead and 12-leads ECG models, we employed a light gradient boosting machine (LGBM) algorithm. This machine learning (ML) algorithm is based on gradient boosting decision trees. LightGBM hyperparameters were optimized via Bayesian optimization using the bayes_opt Python library under patient-level cross-validation, targeting PR-AUC with early stopping. The optimized configuration with the highest mean CV PR-AUC was retained; a baseline untuned LightGBM was trained under identical folds for comparison. No external data were used for model selection (Figure 1).

To preclude information leakage, we performed patient-level splitting so that no recordings from the same individual appeared across development and evaluation folds. All preprocessing (e.g., scaling/filters) was fit on the training fold only and applied unchanged to validation/test data. Hyperparameter tuning and threshold selection were confined to the validation fold within a nested cross-validation scheme; probability calibration (Platt/isotonic) was fitted on training/validation only and not on the test set.

### Statistical analysis

All statistical analyses were performed using R statistical software (version 4.2.1) and Python (version 3.8). Continuous variables are presented as mean values with standard deviations (SDs), while categorical variables are shown as numbers of patients with corresponding percentages. To compare the means of two continuous variables, we employed Student's *t*-test, and for categorical variables, we used Pearson's Chi-square test. Additionally, receiver operating characteristic (ROC) analysis was conducted to assess the performance of the developed ML models. Statistical significance was determined using a two-tailed *p*-value of less than 0.05.

## Results

**Figure 2:**
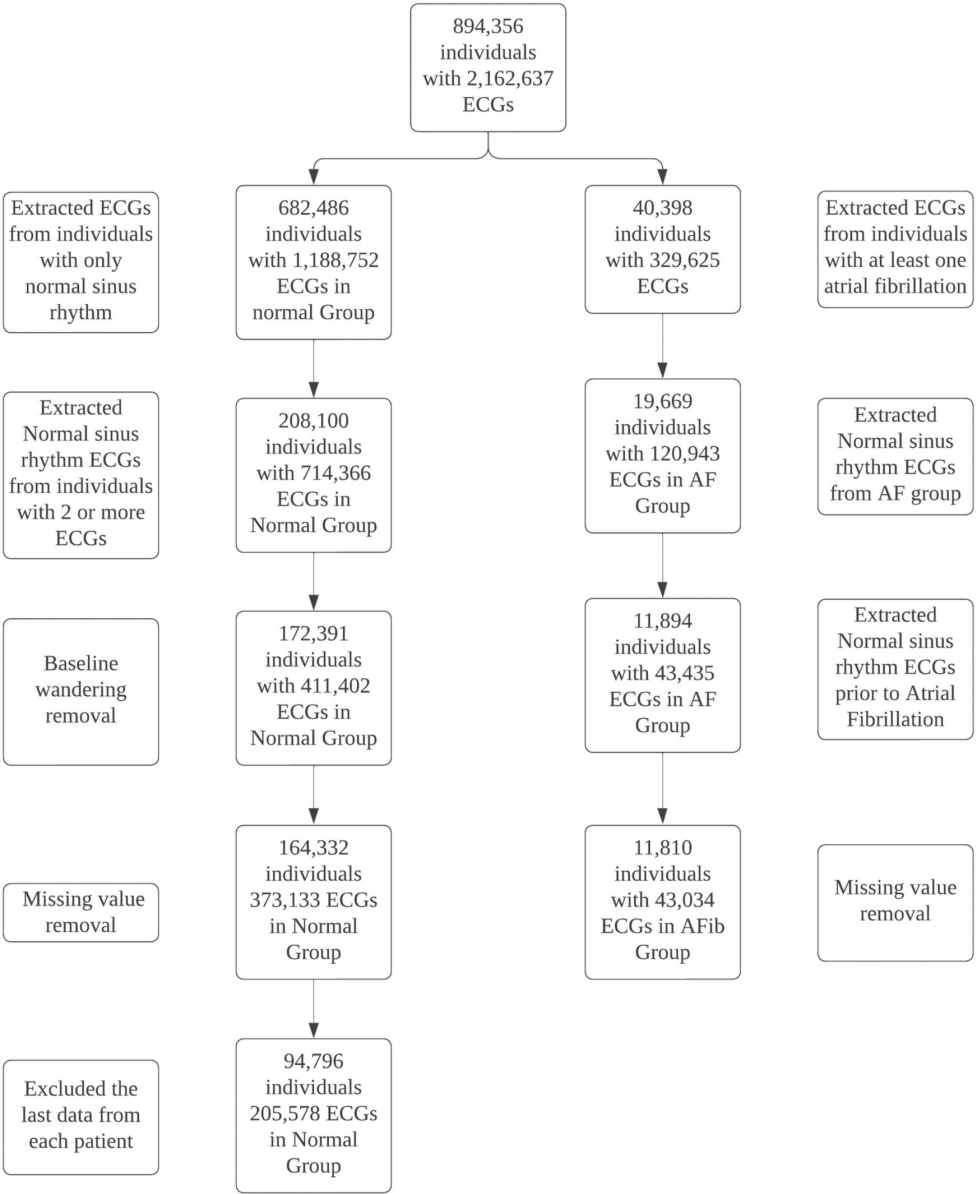
Flow diagram for patients and ECGs selection.

A total of 2,162,637 ECGs were identified from 894,356 adult patients. Out of these, 1,914,025 ECGs from 787,750 patients were excluded based on the study criteria (Figure 2). Ultimately, the machine learning model was trained on 248,612 ECGs from 106,606 patients, of which 11,810 were identified as definite AF cases. The mean age of patients in the normal group was 56.1 ± 13.7 years, while the mean age in the AF group was 65.4 ± 12.8 years (Table 1). Additionally, the proportion of males was significantly higher in the AF group compared to the normal group, with 50.2% of males in the AF group vs. 47.4% in the normal group (*p* < 0.001).

**Table 1:** Baseline characteristics.

Variable	Overall (*n* = 106,606)	NSR group (*n* = 94,796)	AF group (*n* = 11,810)	*P*-value
Age, years	57.0 ± 13.9	56.1 ± 13.7	65.4 ± 12.8	<0.001
Male, *n* (%)	50,902 (47.7)	44,978 (47.4)	5,924 (50.2)	<0.001
Number of ECGs per patient	2.3 ± 2.3	2.2 ± 1.9	3.6 ± 4.2	<0.001

NSR, normal sinus rhythm; AF, atrial fibrillation; ECG, electrocardiogram.

**Figure 3:**
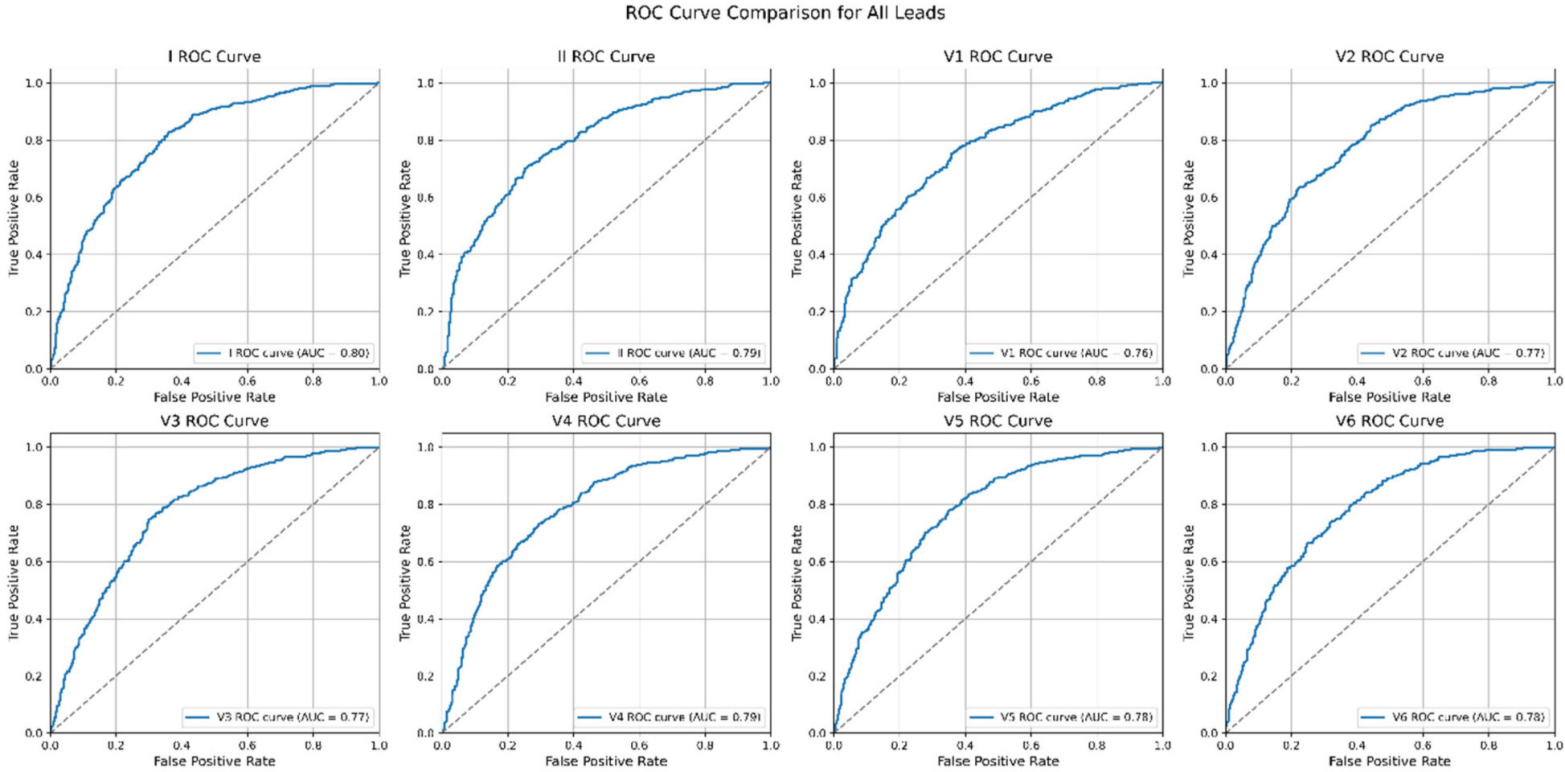
Comparison of AF prediction performance between each single-lead.

The performance of each single-lead model for AF prediction is presented in Table 2. The AUROC (area under the receiver operating characteristic curve) for the single-lead model from lead I was 0.872, indicating it had the highest performance among the nine leads, followed by V6 (0.865) and lead II (0.862) (Table 2 and Figure 3). However, there was no significant difference in the performance of AF prediction across the 9 leads. The results from the external validation were comparable to those from the internal validation. The AUROC for lead I was the highest at 0.801, followed by leads II (0.793) and V4 (0.786) (Table 3).

**Table 2:** Performance of AF prediction of each single lead model (internal validation).

Lead	AUROC	Sensitivity	Specificity	PPV	NPV	F1 score
Lead I	0.872	0.776	0.800	0.448	0.945	0.568
Lead II	0.862	0.764	0.797	0.440	0.942	0.559
Lead III	0.844	0.740	0.783	0.417	0.935	0.533
Lead V1	0.849	0.762	0.774	0.414	0.940	0.536
Lead V2	0.855	0.769	0.780	0.422	0.942	0.545
Lead V3	0.855	0.765	0.780	0.420	0.941	0.543
Lead V4	0.855	0.764	0.785	0.427	0.941	0.548
Lead V5	0.860	0.770	0.788	0.431	0.942	0.553
Lead V6	0.865	0.776	0.786	0.432	0.944	0.555

AF, atrial fibrillation; AUROC, Area Under the Receiver Operating Characteristic Curve; PPV, positive predictive value; NPV, negative predictive value.

**Table 3:** Performance of AF prediction of each single lead models (external validation).

Lead	AUROC	Sensitivity	Specificity	PPV	NPV	F1 score
Lead I	0.801	0.826	0.642	0.698	0.787	0.756
Lead II	0.793	0.702	0.748	0.736	0.715	0.719
Lead V1	0.761	0.754	0.642	0.678	0.723	0.714
Lead V2	0.775	0.632	0.780	0.742	0.679	0.683
Lead V3	0.772	0.748	0.700	0.714	0.735	0.731
Lead V4	0.786	0.732	0.708	0.715	0.725	0.723
Lead V5	0.775	0.776	0.652	0.690	0.744	0.731
Lead V6	0.782	0.736	0.684	0.700	0.722	0.717

AF, atrial fibrillation; AUROC, Area Under the Receiver Operating Characteristic Curve; PPV, positive predictive value; NPV, negative predictive value.

**Figure 4:**
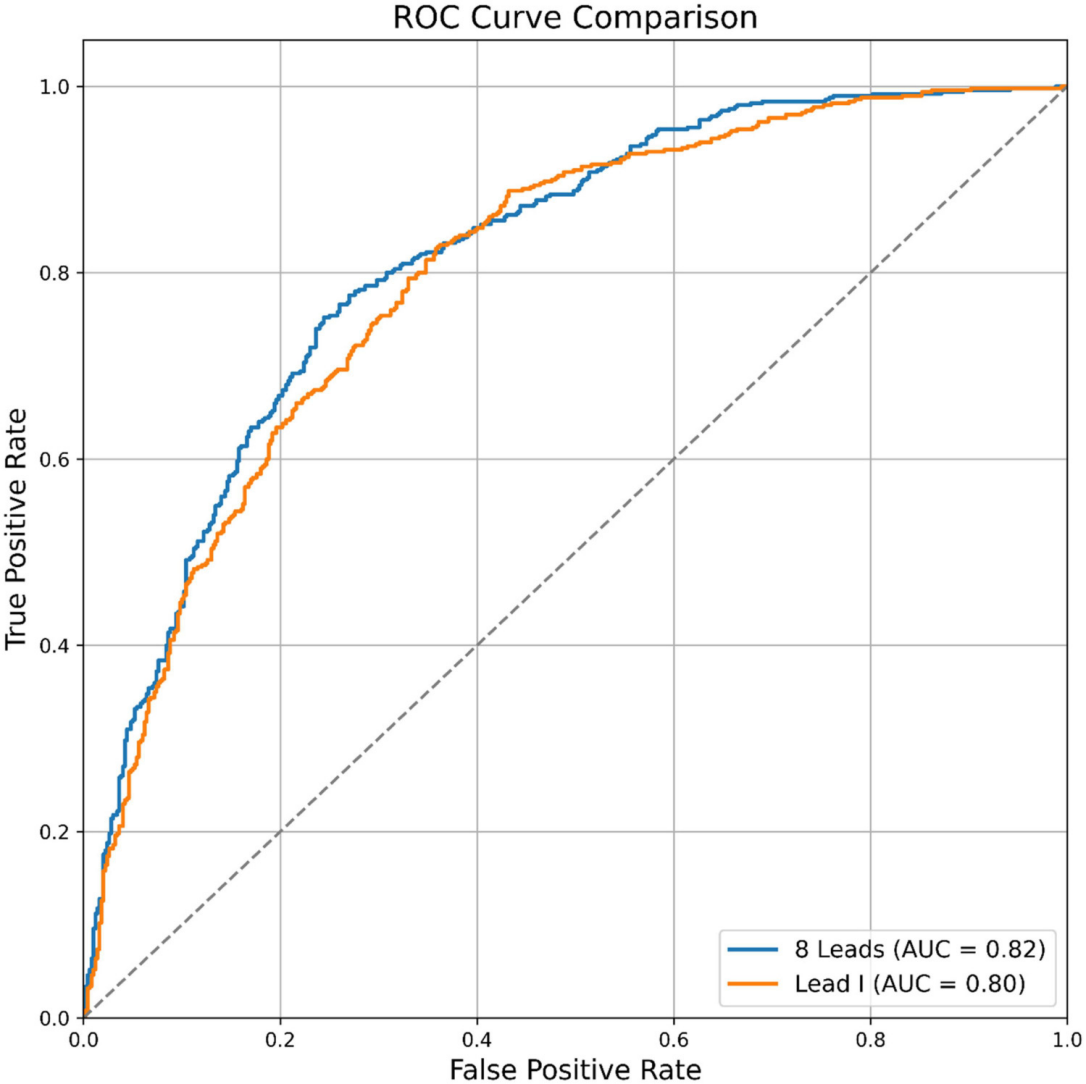
Comparison of AF prediction performance between single-lead and 12-lead.

The AUROC of the single-lead (lead I) model was 0.872, while the AUROC of the 12-lead model was 0.905 (single-lead vs. 12-lead model: sensitivity 0.776 vs. 0.800; specificity 0.800 vs. 0.839; positive predictive value 0.448 vs. 0.510; negative predictive value 0.945 vs. 0.952; F1 score 0.568 vs. 0.623) (Table 4). The external validation results were comparable to internal validation (single-lead vs. 12-lead model: sensitivity 0.826 vs. 0.752; specificity 0.642 vs. 0.756; accuracy 0.820 vs. 0.885; F1 score 0.756 vs. 0.754; AUROC 0.801 vs. 0.816) (Table 4 and Figure 4). The 12-lead model showed a small numerical advantage over the single-lead models, but the differences were modest, and overall performance was broadly comparable.

**Table 4:** Performance of AF prediction of 12-leads ECG model.

Validation cohort	AUROC	Sensitivity	Specificity	PPV	NPV	F1 score
Internal validation	0.905	0.800	0.839	0.510	0.952	0.623
External validation	0.816	0.752	0.756	0.755	0.753	0.754

AF, atrial fibrillation; AUROC, Area Under the Receiver Operating Characteristic Curve; PPV, positive predictive value; NPV, negative predictive value.

## Discussion

We developed ML algorithms for predicting atrial fibrillation using single-lead data derived from 12-lead ECGs. Among the single-lead model developed using each of the nine individual leads, excluding augmented leads, lead I showed the best performance. However, this difference was not significant, and the results were consistent in external validation. Our findings indicate that the AUROC for the single-lead model is 0.801, which is promising, particularly when compared to the 12-lead model that achieved an AUROC of 0.816. Although the performance of the single-lead model is slightly lower, its clinical significance is noteworthy. Single-lead ECGs, commonly available in wearable devices and portable monitors, provide a convenient and accessible method for continuous heart rhythm monitoring. This accessibility is especially important in outpatient settings and for remote patient monitoring, as it allows for real-time assessment and early intervention.

Hygrell et al. demonstrated that a single-lead ECG algorithm, created using a handheld device that measures lead I, can serve as an effective screening tool for predicting AF, especially in population with a wider age distribution (14). Dupulthys et al. developed an AI model incorporating six clinical risk factors alongside a 10-s single-lead ECG, specifically using lead I (15). This single-lead ECG AI algorithm demonstrated performance comparable to that of a 12-lead ECG-based AI model in identifying subclinical AF. Single-lead, handheld intermittent ECG monitoring devices that use lead I are currently widely used. Our research suggested that predicting AF using single lead ECG monitoring based on lead I is feasible.

Population-based screening for AF in individuals over 65 years old, using Holter monitors and single-lead ECGs, has shown a low diagnostic yield (16, 17), and evidence supporting its cost-effectiveness is limited (18, 19). However, AF prediction with the single-lead model can be utilized as a preliminary screening tool to identify patients who need further evaluation. Therefore, selecting patients who require longer ECG monitoring with the single-lead AI model could help reduce healthcare costs while also potentially improving cost-effectiveness. The ultimate goal of AF screening is to reduce preventable stroke events by selecting patients with subclinical or high-risk new-onset AF followed by anticoagulation therapy. Recently, several studies reported that AF screening using novel ambulatory ECG monitoring devices such as Zio Patch, Kardia mobile, and Apple watch resulted in a higher rate of AF diagnosis (20–22). Notably, a microsimulation decision-analytic model showed that AF screening using these wearable devices is cost-effective (23).

The current study has several limitations. First, it excluded three augmented leads (aVR, aVL, and aVF), which could provide additional insights, limiting the analysis to 9 out of the 12 standard leads. Second, performance metrics for the external validation dataset were lower than those for internal validation, indicating potential variations in patient populations or data quality. While the machine learning model was trained on a large dataset of 248,612 ECGs, there is a risk of overfitting due to the model's complexity. Third, we did not employ synthetic oversampling/undersampling. Finally, the study lacks long-term patient follow-up, which is necessary to evaluate the real-world impact on outcomes like atrial fibrillation-related complications. Long-term randomized controlled studies are essential for assessing the effectiveness of early AF detection and intervention.

Future research should aim to improve the performance of single-lead ECG algorithms to make them more comparable to 12-lead models. This may involve incorporating additional features, such as demographic and clinical variables, to increase prediction accuracy. Additionally, long-term large-scale randomized controlled studies that evaluate the impact of single-lead ECG monitoring on patient outcomes and healthcare utilization are crucial for understanding its full potential in clinical practice.

## Conclusion

A single-lead ECG ML model has shown promise in predicting new-onset AF, particularly when using lead I. The performance of the single-lead model is comparable to that of the 12-lead ML model. Implementing this technology in a single-lead ECG patch monitoring device could enhance the screening of AF in the general population.

## Data Availability

The datasets presented in this study can be found in online repositories. The names of the repository/repositories and accession number(s) can be found in the article/Supplementary Material.
